# Elevated Risk of Fluoropyrimidine-Associated Toxicity in European Patients with *DPYD* Genetic Polymorphism: A Systematic Review and Meta-Analysis

**DOI:** 10.3390/jpm12020225

**Published:** 2022-02-06

**Authors:** Woorim Kim, Young-Ah Cho, Dong-Chul Kim, Kyung-Eun Lee

**Affiliations:** 1 College of Pharmacy, Chungbuk National University, Cheongju 28160, Korea; wanppa@hotmail.com; 2 College of Pharmacy, Gyeongsang National University, Jinju 52828, Korea; cyapham@hanmail.net; 3 The Prime Hospital, Jinju 52642, Korea; 4 Department of Pathology, Gyeongsang National University Hospital, Jinju 52727, Korea; 5 School of Medicine, Gyeongsang National University, Jinju 52828, Korea

**Keywords:** fluoropyrimidine, dihydropyrimidine dehydrogenase, *DPYD*, toxicity, meta-analysis

## Abstract

**Background:** Fluoropyrimidine is widely used owing to its clinical efficacy, however, patients with dihydropyrimidine dehydrogenase (DPD) deficiency can experience fluoropyrimidine-associated toxicity. The dihydropyrimidine dehydrogenase (*DPYD*) gene encodes DPD, and studies suggest that *DPYD* polymorphisms can result in DPD deficiency. Since there is not a complete consistency of how much the risk of complication is elevated, we aimed to conduct a systematic literature review and a meta-analysis to provide the risk of fluoropyrimidine-associated toxicity in patients with *DPYD* rs1801160 polymorphism. **Methods:** We searched for qualifying studies published before October 2021 from PubMed, Web of Science, and EMBASE based on Preferred Reporting Items for Systematic Reviews and Meta-Analyses (PRISMA) guidelines. Odds ratios (ORs) and 95% confidence intervals (CIs) were calculated to evaluate the strength of the association between rs1801160 polymorphism and toxicities. A sensitivity analysis using the leave-one-out method was performed on the overall toxicity. **Results:** The pooled OR for overall toxicity in the patients with A allele was elevated 1.73 times higher than those with the GG genotype (95% CI 1.44–2.07). Sensitivity analysis yielded similar results, showing the robustness of the result. Subjects with variants showed a 2.37-fold increased hematological toxicity (95% CI 1.48–3.81); especially a 1.87-fold increased neutropenia compared to patients with wildtype (95% CI 1.49–2.34). Patients with A allele revealed 1.22 times higher gastrointestinal toxicity compared to those with GG genotype (95% CI 0.93–1.61), and among gastrointestinal toxicity, the risk of diarrhea was elevated 1.43 times higher in those with variants than patients with wildtype (95% CI 1.12–1.83). **Conclusions:** rs1801160 polymorphism is associated with elevated fluoropyrimidine-associated toxicity. Therefore, rs1801160 can be a potential candidate for DPD deficiency screening prior to fluoropyrimidine-based regimen.

## 1. Introduction

For several decades, cancer has remained one of the most devastating diseases inflicted on mankind, undoubtedly being one of the leading causes of death. It is expected that by 2040, the number of cancer-related deaths would exceed a stunning 16 million cases [1]. Thus, it is natural that scientists around the world have attempted to tackle this seemingly impregnable ailment, yielding various treatment methods. One such medication class is fluoropyrimidine, an antimetabolite that has become one of the most frequently prescribed chemotherapeutic medications [2]. While higher response rates and modest efficacy have been reported in patients receiving fluoropyrimidine-based regimens [3], the existence of post-therapeutic toxic reactions has raised concerns in the medical community. Such fluoropyrimidine-induced toxicities include gastrointestinal reaction, mucositis, nervous system toxicity, and cardiotoxicity. These complications are not extremely common but may be serious and lead to harmful consequences. Hence, monitoring drug-induced toxicities in patients taking fluoropyrimidine would be prudent and critical.

The dihydropyrimidine dehydrogenase gene (*DPYD*) encodes the dihydropyrimidine dehydrogenase (DPD) enzyme, which plays a vital role in the metabolic catabolism of fluoropyrimidine [4]. Studies have already shown that DPD deficiency can cause severe toxicity in patients receiving fluoropyrimidine-based regimens; failure to degrade fluoropyrimidine may exacerbate side effects including myelosuppression or hand-foot syndrome [5,6]. Since genetic variants in the *DPYD* gene may cause DPD deficiency [7], researchers have previously proposed the usefulness of genetic screening for DPD deficiency in patients undergoing fluoropyrimidine-based therapy. Although many genetic variants in the *DPYD* gene are known to affect DPD activity, specific variants associated with fluoropyrimidine-induced toxicity are still being investigated.

rs1801160 of *DPYD*, a missense single nucleotide polymorphism (SNP), is known to be associated with severe toxicity accompanying fluoropyrimidine-based regimens. Previous researches have shown the association between rs1801160 polymorphism and the aforementioned drug-induced complications; however, the results across studies failed to show consistency. Therefore, the present study aimed to investigate the possible association between rs1801160 polymorphism and fluoropyrimidine-induced toxicity through a systematic literature review and meta-analysis. 

## 2. Methods

### 2.1. Search Strategy

Two investigators independently performed a systematic search for all studies published before 18 October 2021 using PubMed, Web of Science, and EMBASE. The following search terms were included: (DPYD OR DPD OR (dihydropyrimidine dehydrogenase)) AND (polymorph* OR variant* OR mutation* OR genotyp* OR phenotyp* OR haplotyp* OR allele* OR SNP* OR pharmacogen* OR rs1801160 OR Val732Ile OR 2194G>A) AND (toxicity OR adverse OR side-effects OR (adverse effects)). Once duplicates were removed, initial screening through the titles and abstracts were performed to pinpoint potential studies for analysis. Subsequently, a full-text review was carried out to finalize studies to include in this study according to the eligibility criteria.

### 2.2. Inclusion and Exclusion Criteria

Studies were included if they (1) were randomized controlled trials (RCT) or cohort studies; (2) included patients receiving fluoropyrimidine-based regimens; (3) evaluated the association of rs1801160 SNP with toxicity; (4) included applicable data on genotype in both cases and controls; or (5) published in English. Studies were excluded if they were (1) reviews, comments, letters, updates, news, editorials, conference or meeting abstracts, case reports, or case series; (2) in vitro or animal studies; (3) unable to extract genotype; or (5) unable to provide appropriate data.

### 2.3. Data Extraction

The following information was collected from each study: the last name of the first author, year of publication, the number of patients, country, the mean of participants’ age, percentage of female subjects, cancer type, treatment regimen, the definition of toxicity, and genotype.

### 2.4. Quality Assessment

The methodologic quality of the selected studies was evaluated using the Newcastle–Ottawa Scale (NOS). The NOS contains three components (subject selection; comparability of study groups; and exposure or outcome), and each study can attain a maximum score of 9.

### 2.5. Statistical Analysis

The odds ratio (OR) with 95% confidence intervals (CIs) was calculated to evaluate the association between toxicity and polymorphism. I^2^ was used for heterogeneity evaluation among studies. When heterogeneity was high (I^2^ > 50%), the random-effects model was applied. If I^2^ was less than or equal to 50%, the fixed-effects model was applied. 

To identify publication bias, both Egger’s and Begg’s regression tests of the funnel plot were generated. A *p*-value less than 0.05 was considered statistically significant. Statistical analyses and publication bias were performed using Review Manager (RevMan) version 5.4 (The Cochrane Collaboration, Copenhagen, Denmark) and RStudio software (version 4.0.0; RStudio: Integrated Development for R, Boston, MA). This meta-analysis was written based on the Preferred Reporting Items for Systematic Reviews and Meta-Analyses (PRISMA) guidelines.

## 3. Results

**Figure 1:**
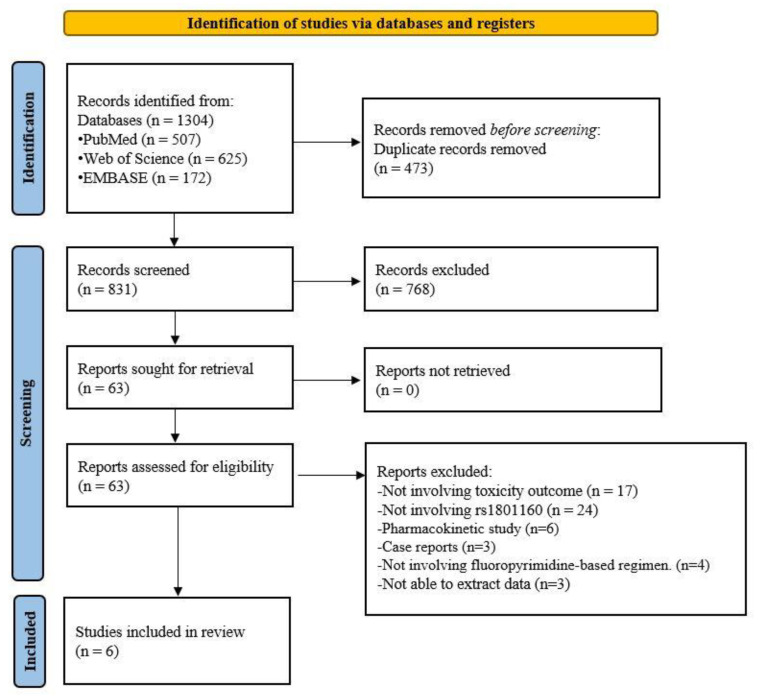
Flow diagram of the study selection process.

A flow diagram of the literature search and selection process is depicted in Figure 1. A total of 1304 records were initially searched from PubMed (*n* = 507), Web of Science (*n* = 625), and EMBASE (*n* = 172). After excluding duplicates (*n* = 473) and irrelevant studies (*n* = 768), 63 records were selected for full-text review, and 57 records were excluded due to the following reasons: not related to toxicity outcome (*n* = 17), not related to polymorphisms of rs18001160 (*n* = 24), pharmacokinetic studies (*n* = 6), case reports (*n* = 3), not involving fluoropyrimidine-based regimens (*n* = 4), or unable to extract data (*n* = 3). Ultimately, six studies were included in this meta-analysis [8,9,10,11,12,13]. 

The baseline characteristics of the included studies are displayed in Table 1. The studies were published between 2009 and 2019, and all of them were conducted in Europe. Patients with various cancer types, including colorectal, breast, and gastric cancers, were included in this study. Studies with the fluoropyrimidine-based regimen including FOLFOX4 (5-fluorouracil, leucovorin, and oxaliplatin) were evaluated.

**Figure 2:**
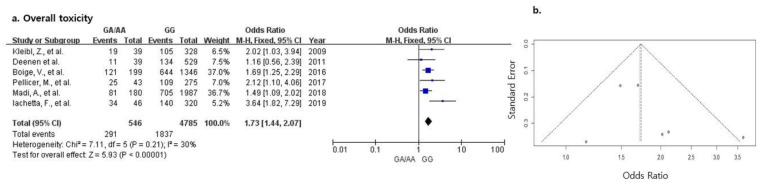
(**a**). Forest plot of the association between fluoropyrimidine-induced overall toxicity and *DPYD* polymorphism (**b**). Funnel plot of fluoropyrimidine-induced overall toxicity and *DPYD* polymorphism.

Figure 2a shows the ORs with 95% CIs for the association between the rs18001160 polymorphism in the *DPYD* gene and the risk for overall fluoropyrimidine-associated toxicity. Overall meta-analysis with six studies indicated that the rs18001160 polymorphism was associated with increased toxicity (OR 1.73, 95% CI 1.44–2.07, *p* < 0.001). Since there was no heterogeneity between the included studies (I^2^ = 30%), the fixed-effects model was used to calculate the effect size. The funnel plot was symmetrical (Figure 2b) and Egger’s and Begg’s tests did not show evidence for publication bias in this meta-analysis (Egger’s test, *p* = 0.352; Begg’s test, *p* = 0.573). The sensitivity analysis showed similar results, suggesting that no one study predominantly affected the pooled results (Table 2).

**Figure 3:**
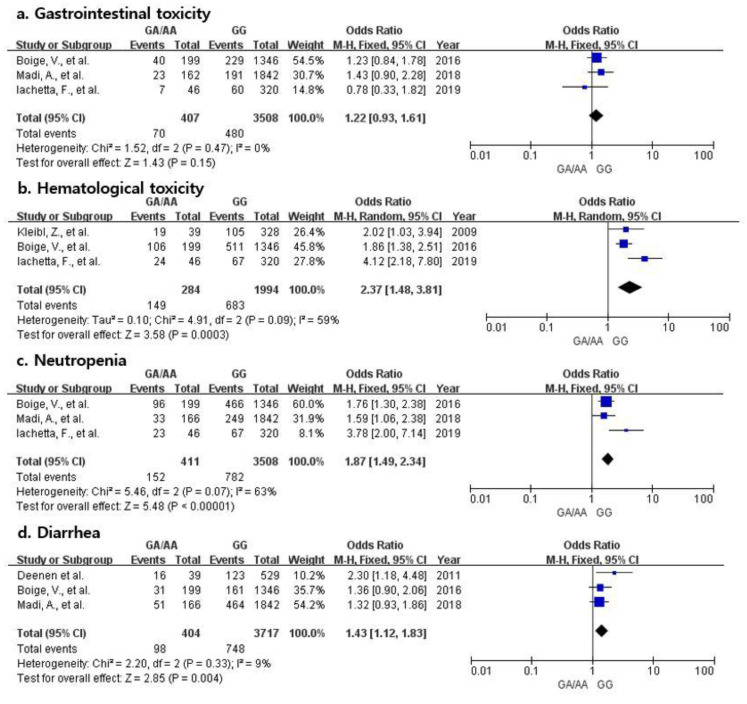
Forest plots of the association between fluoropyrimidine-induced toxicities and *DPYD* polymorphism: (**a**). gastrointestinal toxicity; (**b**). hematological toxicity; (**c**). neutropenia; (**d**). diarrhea.

For specific toxicity analyses, gastrointestinal toxicity, hematological toxicity, neutropenia, and diarrhea were analyzed. As presented in Figure 3, hematological toxicity, neutropenia, and diarrhea involved three studies and suggested that the rs18001160 polymorphism was associated with approximately 2.4-, 1.9-, and 1.4-fold elevated toxicities, respectively (95% CI 1.48–3.81, 1.49–2.34, and 1.12–1.83, respectively).

## 4. Discussion

The main finding of this study is that subjects with the A allele of rs1801160 showed an approximately 1.72-times elevated risk of fluoropyrimidine-associated overall toxicity compared to those with the GG genotype (95% CI 1.44–2.07, *p* < 0.01). Neither Egger’s test nor Begg’s test showed significant publication bias (Egger’s test, *p* = 0.352; Begg’s test, *p* = 0.573). The sensitivity analysis yielded similar results, indicating that the results were robust. Specific types of toxicity, including gastrointestinal toxicity, hematological toxicity, neutropenia, and diarrhea, showed the same trend with ORs of 1.22, 2.37, 1.87, and 1.43, respectively.

About 1% to 3% of the fluoropyrimidine metabolite is cytotoxic; fluorodeoxyuridine monophosphate forms a ternary complex with thymidylate synthase and 5,10-methylene tetrahydrofolate, resulting in the inhibition of DNA synthesis. DPD is known to convert approximately 80% to 85% of fluoropyrimidine into inactive dihydrofluorouracil (DHFU) through hepatic metabolism [14]; this is the rate-limiting step in fluoropyrimidine metabolism. DHFU is then converted to fluoro-β-ureidopropionate by dihydropyrimidinease and further changed to fluoro-β-alanine by β-ureidopropionase [15,16]. For those with DPD deficiency, inadequate metabolism and the subsequently reduced inactivation of fluoropyrimidine may result in fatal toxicity [17]. Several meta-analyses were conducted to investigate associations between *DPYD* polymorphisms and the complications in patients undergoing fluoropyrimidine-based regimens. For instance, Meulendijks et al. showed that *DPYD* polymorphisms rs55886062, rs75017182, and rs56038477 were associated with an increased risk of fluoropyrimidine-associated toxicity [18]. In addition, Terrazzino et al. confirmed that polymorphisms rs3918290 and rs67376798 were risk factors for the development of complications in patients on fluoropyrimidine-based therapy [19]. 

The prevalence of partial and complete DPD deficiency range from 3 to 15% and 0.1 to 0.5%, respectively [20]. Currently, there is no mandatory DPD deficiency screening prior to fluoropyrimidine-based therapy; however, studies including those by the Group of Clinical Pharmacology in Oncology (GPCO)-UNICANCER and the French Network of Pharmacogenetics (RNPGx) recommend screening for DPD deficiency before fluoropyrimidine-based regimens to prevent the various aforementioned complications [21]. Therefore, it is of paramount importance to further evaluate the association between SNPs of DPD-related genes and fluoropyrimidine-induced complications via meta-analyses, increasing the number of observations and statistical power.

rs1801160 is a missense SNP in the coding region of the *DPYD* gene which can affect DPD activity [22]. The minor allele frequencies of rs1801160 are 0.07 in Americans, 0.05 in Europeans, 0.03 in Africans, and 0.02 in Asians [23,24]. Many studies have examined the association between the rs1801160 polymorphism in *DPYD* and fluoropyrimidine-associated toxicity. However, the risk of overall complications varied from 1.16-fold to 3.64-fold among studies. Li et al. conducted a meta-analysis in 2014 on several SNPs, including rs1801160; however, only 2 studies with 628 subjects were included for the analysis. Our meta-analysis updated those results, using 6 studies including more recent data from a total of 5331 subjects. 

This study showed that patients with the A allele had an approximately 2.4- and 1.9-fold increased risk of hematological toxicity and neutropenia, respectively, compared to those with the GG genotype. Hematological toxicity is one of the reasons for chemotherapy discontinuation [25], while neutropenia is a fatal complication in chemotherapy [26]. These toxicities can be life-threatening to the patients and may require dose reduction or treatment delay, resulting in unfavorable clinical outcomes. In this context, this study provided a pooled estimate of fluoropyrimidine-associated toxicity risk in patients with the A allele of rs1801160, opening possibilities of clinical modifications based on genotypic profiles regarding fluoropyrimidine therapy.

This meta-analysis bears several limitations. First, all studies included in this meta-analysis were conducted in the European population. As the allele frequencies of the variants in Europeans (5%) are different from the variants in Asians (2%), further investigation should be assessed to generalize the present results to other ethnic groups. Second, due to limited data availability, critical toxicities including cardiotoxicity could not be analyzed. Third, some confounding factors that could affect the risk of toxicities could not be adjusted for. In general, cancer patients generally take more than one class of medication; hence, co-medications may have affected the study results. Furthermore, while tests did not show evidence for publication bias, the possibility cannot be ruled out due to the low sample size. Nevertheless, this study provided a candidate SNP for screening to prevent fluoropyrimidine-induced complications through an improved estimate of the effect size of the association. As a systematic review and meta-analysis on the rs1801160 polymorphism and fluoropyrimidine-associated toxicity, this analysis may provide clinical evidence for future considerations in the treatment and management of fluoropyrimidine-based regimens.

## Figures and Tables

**Table 1:** Characteristics of studies included.

Authors	Number of Patients	Country	Age (Years)	Female (%)	Cancer Type	Treatment Regimen	Definition of Outcome	Total NOS
Kleibl, 2009 [8]	124	Czech Republic	N/A	N/A	orofacial, esophageal, gastric, cololectal, biliary, pancreatic, pharyngeal, or breast cancer	fluoropyrimidine-based regimens	NCI-CTCAE	7
Deenen, 2011 [9]	568	Netherlands	median 63	39%	advanced colorectal cancer	fluoropyrimidine-based regimens	NCI-CTCAE, version 3.0	7
Boige, 2016 [10]	2559	Multiple sites in Europe	median 60	42.40%	resected stage III colorectal cancer	FOLFOX4 every 2 weeks (1 cycle) with (armB) or without (arm A) cetuximab	NCI-CTCAE, version 3.0	7
Pellicer, 2017 [11]	319	Spain	median 65	47.30%	colorectal cancer	a capecitabine-containing regimen	NCI-CTCAE, version 4.0	7
Madi, 2018 [12]	2183	UK and Ireland	N/A	N/A	advanced colorectal cancer	fluoropyrimidine-based regimens	N/A	7
Iachetta, 2019 [13]	366	Italy	N/A	N/A	colon, gastric, rectum, pancreas, anus, breast, esophagus, bile duct, head and neck, uterine cervix, or valvular cancer	fluoropyrimidine-based regimens	NCI-CTCAE, version 4.0	7

UK: United Kingdom; FOLFOX4: 5-fluorouracil, leucovorin, and oxaliplatin; NCI-CTCAE: national cancer institute-common terminology criteria for adverse events; NOS: Newcastle–Ottawa scale.

**Table 2:** Sensitivity analysis by sequentially excluding each study from the meta-analysis.

Excluded Study	Heterogeneity I^2^ (%)	Statistical Model	Odds Ratio (95% CI)
None	30	Fixed	1.73 (1.44, 2.07)
Kleibl, 2009	42	Fixed	1.71 [1.42, 2.06]
Deenen, 2011	32	Fixed	1.78 [1.47, 2.14]
Boige, 2016	44	Fixed	1.75 [1.40, 2.19]
Pellicer, 2017	40	Fixed	1.70 [1.41, 2.05]
Madi, 2018	31	Fixed	1.49 [1.09, 2.02]
Iachetta, 2019	0	Fixed	1.62 [1.34, 1.96]

CI: confidence interval.

## Data Availability

Not applicable.

## References

[1] Institute NC Cancer Statistics: National Institute of Health. https://www.cancer.gov/about-cancer/understanding/statistics.

[2] Institute NC NCI Dictionary of Cancer Terms: National Institute of Health. https://www.cancer.gov/publications/dictionaries/cancer-terms/.

[3] Kim B.J., Yoo C., Kim K.-P., Hyung J., Park S.J., Ryoo B.-Y., Chang H.-M. (2017). Efficacy of fluoropyrimidine-based chemotherapy in patients with advanced biliary tract cancer after failure of gemcitabine plus cisplatin: Retrospective analysis of 321 patients. Br. J. Cancer.

[4] Liu X.-Q., Zhuang M., Wang Z., Huber R.M. (2014). Correlation between dihydropyrimidine dehydrogenase and efficacy and toxicity of fluoropyrimidine drugs. Eur. Rev. Med. Pharmacol. Sci..

[5] Boisdron-Celle M., Morel A., Gamelin E. (2010). Dihydropyrimidine dehydrogenase deficiency and toxicity to fluoropyrimidine. Ann. Biol. Clin..

[6] Lampropoulou D.I., Laschos K., Amylidi A.-L., Angelaki A., Soupos N., Boumpoucheropoulos S., Papadopoulou E., Nanou E., Zidianakis V., Nasioulas G. (2020). Fluoropyrimidine-induced toxicity and DPD deficiency. A case report of early onset, lethal capecitabine-induced toxicity and mini review of the literature. Uridine triacetate: Efficacy and safety as an antidote. Is it accessible outside USA?. J. Oncol. Pharm. Pract..

[7] van Staveren M.C., Guchelaar H.J., van Kuilenburg A.B., Gelderblom H., Maring J.G. (2013). Evaluation of predictive tests for screening for dihydropyrimidine dehydrogenase deficiency. Pharm. J..

[8] Kleibl Z., Fidlerova J., Kleiblova P., Kormunda S., Bilek M., Bouskova K., Sevcik J., Novotny J. (2009). Influence of dihydropyrimidine dehydrogenase gene (DPYD) coding sequence variants on the development of fluoropyrimidine-related toxicity in patients with high-grade toxicity and patients with excellent tolerance of fluoropyrimidine-based chemotherapy. Neoplasma.

[9] Deenen M.J., Tol J., Burylo A.M., Doodeman V.D., De Boer A., Vincent A., Guchelaar H.-J., Smits P.H., Beijnen J.H., Punt C.J. (2011). Relationship between Single Nucleotide Polymorphisms and Haplotypes in DPYD and Toxicity and Efficacy of Capecitabine in Advanced Colorectal Cancer. Clin. Cancer Res..

[10] Boige V., Vincent M., Alexandre P., Tejpar S., Landolfi S., le Malicot K., Greil R., jan Cuyle P., Yilmaz M., Faroux R. (2016). DPYD Genotyping to Predict Adverse Events Following Treatment With Fluorouracil-Based Adjuvant Chemotherapy in Patients With Stage III Colon Cancer: A Secondary Analysis of the PETACC-8 Randomized Clinical Trial. JAMA Oncol..

[11] Pellicer M., García-González X., García M.I., Robles L., Grávalos C., García-Alfonso P., Pachón V., Longo F., Martínez V., Blanco C. (2017). Identification of new SNPs associated with severe toxicity to capecitabine. Pharmacol. Res..

[12] Madi A., Fisher D., Maughan T.S., Colley J.P., Meade A.M., Maynard J., Humphreys V., Wasan H., Adams R.A., Idziaszczyk S. (2018). Pharmacogenetic analyses of 2183 patients with advanced colorectal cancer; potential role for common dihydropyrimidine dehydrogenase variants in toxicity to chemotherapy. Eur. J. Cancer.

[13] Iachetta F., Bonelli C., Romagnani A., Zamponi R., Tofani L., Farnetti E., Nicoli D., Damato A., Banzi M., Casali B. (2019). The clinical relevance of multiple DPYD polymorphisms on patients candidate for fluoropyrimidine based-chemotherapy. An Italian case-control study. Br. J. Cancer.

[14] Merloni F., Ranallo N., Scortichini L., Giampieri R., Berardi R. (2019). Tailored therapy in patients treated with fluoropyrimidines: Focus on the role of dihydropyrimidine dehydrogenase. Cancer Drug Resist..

[15] Kopper L., Lapis K., Institóris L. (1976). Incorporation of 3H-dibromodulcitol and 3H-dianhydrodulcitol into ascites tumor cells. Autoradiographic study. Neoplasma.

[16] van Kuilenburg A.B.P., Meinsma R., Zonnenberg B.A., Zoetekouw L., Baas F., Matsuda K., Tamaki N., van Gennip A.H. (2003). Dihydropyrimidinase deficiency and severe 5-fluorouracil toxicity. Clin. Cancer Res..

[17] Amstutz U., Froehlich T.K., Largiadèr C.R. (2011). Dihydropyrimidine dehydrogenase gene as a major predictor of severe 5-fluorouracil toxicity. Pharmacogenomics.

[18] Meulendijks D., Henricks L., Sonke G., Deenen M.J., Froehlich T.K., Amstutz U., Largiader C., Jennings B., Marinaki A.M., Sanderson J.D. (2015). Clinical relevance of DPYD variants c.1679T>G, c.1236G>A/HapB3, and c.1601G>A as predictors of severe fluoropyrimidine-associated toxicity: A systematic review and meta-analysis of individual patient data. Lancet Oncol..

[19] Terrazzino S., Cargnin S., Del Re M., Danesi R., Canonico P.L., Genazzani A.A. (2013). DPYD IVS14+1G>A and 2846A>T genotyping for the prediction of severe fluoropyrimidine-related toxicity: A meta-analysis. Pharmacogenomics.

[20] Loriot M.A., Ciccolini J., Thomas F., Barin-Le-Guellec C., Royer B., Milano G., Picard N., Becquemont L., Verstuyft C., Narjoz C. (2018). Dihydropyrimidine déhydrogenase (DPD) deficiency screening and securing of fluoropyrimidine-based chemotherapies: Update and recommendations of the French GPCO-Unicancer and RNPGx networks. Bull. Cancer.

[21] Abdullah-Koolmees H., van Keulen A.M., Nijenhuis M., Deneer V.H.M. (2020). Pharmacogenetics Guidelines: Overview and Comparison of the DPWG, CPIC, CPNDS, and RNPGx Guidelines. Front. Pharmacol..

[22] Zhang X.-P., Bai Z.-B., Chen B.-A., Feng J.-F., Yan F., Jiang Z., Zhong Y.-J., Wu J.-Z., Chen L., Lu Z.-H. (2012). Polymorphisms of dihydropyrimidine dehydrogenase gene and clinical outcomes of gastric cancer patients treated with fluorouracil-based adjuvant chemotherapy in Chinese population. Chin. Med. J..

[23] Phan L., Jin Y., Zhang H., Qiang W., Shekhtman E., Shao D., Revoe D., Villamarin R., Ivanchenko E., Kimura M. (2020). ALFA: Allele Frequency Aggregator: National Center for Biotechnology Information, U.S. National Library of Medicine. www.ncbi.nlm.nih.gov/snp/docs/gsr/alfa/.

[24] Ward L.D., Kellis M. (2012). HaploReg: A resource for exploring chromatin states, conservation, and regulatory motif alterations within sets of genetically linked variants. Nucleic Acids Res..

[25] Ouyang Z., Peng D., Dhakal D.P. (2013). Risk factors for hematological toxicity of chemotherapy for bone and soft tissue sarcoma. Oncol. Lett..

[26] Ba Y., Shi Y., Jiang W., Feng J., Cheng Y., Xiao L., Zhang Q., Qiu W., Xu B., Xu R. (2020). Current management of chemotherapy-induced neutropenia in adults: Key points and new challenges: Committee of Neoplastic Supportive-Care (CONS), China Anti-Cancer Association Committee of Clinical Chemotherapy, China Anti-Cancer Association. Cancer Biol. Med..

